# The Ferroptosis Inhibitor Liproxstatin-1 Ameliorates LPS-Induced Cognitive Impairment in Mice

**DOI:** 10.3390/nu14214599

**Published:** 2022-11-01

**Authors:** Yang Li, Miao Sun, Fuyang Cao, Yu Chen, Linlin Zhang, Hao Li, Jiangbei Cao, Jie Song, Yulong Ma, Weidong Mi, Xiaoying Zhang

**Affiliations:** 1 Chinese PLA Medical School, Beijing 100853, China; 2 Department of Anesthesiology, First Medical Center of Chinese PLA General Hospital, Beijing 100853, China; 3 Department of Anesthesiology, Sixth Medical Center of Chinese PLA General Hospital, Beijing 100048, China; 4 Department of Anesthesiology, Tianjin Medical University General Hospital, Tianjin 300052, China; 5 Nursing Department, First Medical Center of Chinese PLA General Hospital, Beijing 100853, China

**Keywords:** ferroptosis, iron, neuroinflammation, memory impairment, oxidative stress

## Abstract

CNS inflammation is known to be an important pathogenetic mechanism of perioperative neurocognitive disorder (PND), and iron overload was reported to participate in this process accompanied by oxidative stress. Ferroptosis is an iron-dependent form of cell death, and occurs in multiple neurodegenerative diseases with cognitive disorder. However, the effect of ferroptosis in inflammation-related PND is unknown. In this study, we found that the ferroptosis inhibitor liproxstatin-1 ameliorated memory deficits in the mouse model of lipopolysaccharide (LPS)-induced cognitive impairment. Moreover, liproxstatin-1 decreased the activation of microglia and the release of interleukin (IL)-6 and tumor necrosis factor-alpha (TNF)-α, attenuated oxidative stress and lipid peroxidation, and further weakened mitochondrial injury and neuronal damage after LPS exposure. Additionally, the protective effect of liproxstatin-1 was related to the alleviation of iron deposition and the regulation of the ferroptosis-related protein family TF, xCT, Fth, Gpx4, and FtMt. These findings enhance our understanding of inflammation-involved cognitive dysfunction and shed light on future preclinical studies.

## 1. Introduction

Perioperative neurocognitive disorder (PND), which includes postoperative delirium and postoperative cognitive dysfunction (POCD), is a major challenge facing our rapidly aging population. PND is characterized by impairments in cognitive domains, such as memory, attention, and concentration after surgery [1], and is a risk factor for significant complications, such as dementia and even death [2,3]. The surgery-induced central inflammatory response is closely related to PND in both humans and animal models. Massive evidence showed that major surgeries could induce a widespread systemic inflammatory response, and multiple biomarkers of neuro-inflammatory response were widely observed to have an association to the pathophysiological changes underpinning POCD and cognitive deficits in clinical studies [4,5]. This conclusion was verified in multiple surgery rodent models in terms of its mechanism, showing that the upregulation of pro-inflammatory cytokines and inflammatory mediators in the central nervous system (CNS) could cause obvious cognitive impairment in animals, and that it could be blocked by anti-inflammatory agents [6,7,8]. Inflammation leads to oxidative stress, contributes to mitochondrial dysfunction [9,10,11,12], and induces a variety of metabolic disorders, such as iron metabolism disorders. In turn, iron deposition plays a role in cognitive impairment-related diseases. Our previous study showed that oxidative stress and iron accumulation play a significant role in the mouse model of lipopolysaccharide (LPS)-induced memory impairment [13]. However, the precise pathogenetic mechanism underlying the effect of neuroinflammation on cognitive function, and the means by which iron regulates this process, have so far not been fully stated.

Ferroptosis, characterized by dysfunctional iron metabolism and lipid peroxidation, is a newly discovered form of cell death. Unlike other forms of cell death, such as necrosis, apoptosis, and autophagy, ferroptosis possesses unique morphological, biochemical, and genetic features [14]. Emerging evidence shows that ferroptosis occurs in multiple neurological diseases with cognitive disorder, including Parkinson’s disease, stroke, and Huntington’s disease [15]. Further, its occurrence is accompanied by dysfunctional iron metabolism, mitochondrial dysfunction, lipid peroxidation, and glutathione peroxidase 4 (Gpx4) downregulation [16,17,18,19,20]. Our previous study revealed that LPS-induced memory impairments are associated with abnormal iron metabolism in mice. Further, the iron chelator deferoxamine can prevent these neuroinflammation-induced impairments in learning and memory by preventing iron accumulation and alleviating oxidative stress [13]. In addition, ferroptosis inhibitors, such as liproxstatin-1 (Lip-1) and ferrostatin-1, have shown protective effects on neurons and ameliorate cognitive dysfunction in animal models of neurodegenerative diseases [16,21]. Even so, little is known about whether ferroptosis takes part in the neuroinflammation-induced cognitive impairment, let alone the mechanism by which it participates.

Accordingly, we hypothesized that ferroptosis is involved in the occurrence of cognitive impairment induced by inflammation in the central nervous system. To examine this hypothesis, we assessed the effect of the ferroptosis inhibitor liproxstatin-1 on LPS-induced neuroinflammation and cognitive performance in mice to explore potential ferroptosis-targeted treatment strategies for PND.

## 2. Materials and Methods

### 2.1. Animals

Two hundred and sixteen male C57BL/6 mice aged 8 weeks were obtained from the Beijing SPF Animal Technology Company (Beijing, China; Permit number SCXK(Jing) 2019-0010). The mice were housed under standard conditions in a temperature- and humidity-controlled environment and had free access to food and water. All experiments were performed one week after the mice were acclimatized to the environment. All animal procedures in the experiment strictly complied with the guidelines of the Animal Welfare Ethics Committee of Chinese PLA General Hospital. The 216 mice were randomly divided into four groups (*n* = 54 per group): CON group (aCSF + vehicle), LPS group (LPS + vehicle), Lip-1 group (aCSF + liproxstatin-1), and LPS + Lip-1 group (LPS + Liproxstatin-1). Mice were treated with intraperitoneal injections of liproxstatin-1 (10 mg/kg, dissolved in PBS with 10% DMSO; MedChem-Express, Monmouth Junction, NJ, USA) or an equal volume of vehicle once daily during the experiment. Five days after the Morris Water Maze (MWM) learning phase, LPS was injected intracerebroventricularly (2 μg LPS dissolved in 2 μL artificial cerebrospinal fluid [aCSF, containing 140 mM NaCl, 3.0 mM KCl, 2.5 mM CaCl_2_, 1.2 mM Na_2_HPO, and 41.0 mM MgCl_2_]). The vehicle groups received an equal volume of aCSF.

Six hours after LPS administration, five mice from each group were randomly chosen and sacrificed. Hippocampal tissue was obtained to examine cytokine levels, according to our previous study [22]. The remaining 49 mice in each group were used for four behavioral tests, of which, 25 mice were used for the MWM test, 12 mice for both the open field (OF) test and the new object recognition (NOR) test, and another 12 mice for the contextual fear conditioning (FC) test. All the behavioral tests were performed 24 h after LPS administration. All 12 mice in both the NOR and OF test groups, as well as 8 random mice in each group of the MWM probe test group were sacrificed for molecular measurements once after the behavioral tests were finished 24 h after LPS microinjection, and the remaining 17 mice in the MWM test group proceeded with the working memory test for three consecutive days. To avoid the effect of electric shock, the mice that finished the FC test were not used for any molecular tests.

### 2.2. Intracerebroventricular Microinjection Surgery

For the stereotactic injection of LPS or aCSF, the mice were placed on a stereotactic apparatus after being anesthetized with Avertin (200 mg/kg, i.p.). Using specific coordinates (posterior: 0.5, lateral: ±1.0, and ventral: 2.0 from the bregma [in mm]), the lateral ventricle was located. LPS or aCSF was injected at a uniform speed within 3 min, and the needle was left in place for at least 3 min after injection.

### 2.3. Open Field Test

In order to eliminate differential interference from the spontaneous activity of the mice, the OF test was performed 24 h after LPS (or aCSF) microinjection treatment. The square base of the test chamber was divided into 16 equal areas, and the mice were placed in the same corner and allowed to freely explore for 2 min. The count of line crossings and the total distance were recorded in the next 8 min. The open field was cleaned with 75% ethanol and dried after each mouse finished the test.

### 2.4. Morris Water Maze Test

The MWM test is used to evaluate spatial learning and memory in mice. For this test, a circular swimming pool (diameter = 122 cm) was filled with water to a depth of 30 cm. The temperature of the water was 22 ± 1 °C. The pool was housed in a visually cued room divided into four separate quadrants. The movement of the animals was recorded by cameras. Spatial learning was assessed through five consecutive days of training. The mice had to rely on surrounding cues to navigate from the initial location and find a submerged escape platform (10 cm^2^). At the beginning of the test, the mice were released into the water at the middle point of one quadrant facing the pool wall. During each trial, the mouse had to find the platform within 60 s. If it failed to do so, it was picked up and placed on the platform for 15 s to allow it to memorize the platform’s location. Subsequently, the mouse was dried and placed back in its cage, and the next mouse would begin the training trial. After all the animals finished trial 1, trials 2, 3, and 4 were performed, until all mice had completed four trials each day for five consecutive days. On the sixth day, the mice received LPS (or aCSF) microinjection. Twenty-four hours after LPS (or aCSF) microinjection, the probe test was performed to access the reference memory of the mice in the absence of the platform. Each mouse started from the quadrant opposite to the platform and had to swim for 60 s. The swimming speed, number of platform site crossings, percentage of distance and time within the target quadrant, and escape latency were recorded.

Eight mice in each group were sacrificed for molecular measurements 24 h post LPS microinjection after the probe test, and the remaining 17 mice underwent the working memory trial on days 1 to 3 after LPS (or aCSF) microinjection. The platform and mice were randomly repositioned to evaluate working-dependent learning and memory. The mice underwent two trials per day for three consecutive days, and had to learn the new position of the platform during the first training trial. The second test trial was performed after 15 s, and the experimental procedure was the same as in the first trial. If a mouse recalled the training, it swam a shorter distance to find the platform during the test trial. The platform was moved daily; hence, the mice could not apply the learned location of the platform from the previous day. Therefore, the recalling during the test trial was dependent on the training trial of that very day. Hence, only temporary or working memory was measured. The escape latency to the platform in the test trial was recorded as a measure of the temporary or working memory. In the test trial, the escape latency was assumed to be 60 s if the mice could not reach the platform within 60 s. Liproxstatin-1 treatment was administered 2 h before the daily behavioral test.

### 2.5. New Object Recognition Test

The NOR test was performed as previously described [22,23]. On the first day, the mouse was placed in an empty square chamber to freely explore for 5 min. Twenty-four hours after habituation, the mouse was placed in the same chamber as the first day, with two of the same objects in the back left and right corners of the chamber, each 8 cm away from the sidewalls. The mouse was placed at the middle of the chamber opposite the two same objects and allowed to explore for 8 min to become acquainted with the objects. After the training phase, each mouse received an LPS intracerebroventricular injection and proceeded to the NOR test 24 h after. The mouse was placed in the same chamber as the training phase, but one of the old objects was replaced by a novel object of the same size, but with a different color and shape. The mouse was allowed to explore the objects for 8 min ad libitum. The apparatus was cleaned with the 75% ethanol after each mouse finished the test. The old/new object exploration was defined as contacting with the objects or directing them with the nose at a distance ≤2 cm. The time taken to explore the familiar object (TF) and the novel object (TN) were recorded. The recognition index was expressed by the ratio TF/(TN + TF).

### 2.6. Contextual Fear Conditioning Test

The contextual fear conditioning test assessed context-dependent learning and memory [24]. In the fear conditioning training session, the mouse was placed into the chamber and allowed to freely explore for 2 min to habituate the context. After habituation, the mouse received a conditioned stimulus three times (CS: 30 s, 80 dB tone stimulus) with a co-terminating unconditioned stimulus (US: electric foot shock of 0.5 mA for 2 s) at a 120 s interval, then was returned to its home cage 2 min after the last shock. The chamber was cleaned with 75% ethanol after each test. On the second day, each mouse received an LPS microinjection. The contextual memory test was conducted 24 h after LPS microinjection. The mice were returned to the very same chamber as the training session, without tone or electric shock for 6 min. The freezing time was recorded to evaluate consolidated fear memory.

### 2.7. Tissue Sampling

The hippocampus is one of the most important regions for learning and memory, as well as being a significant area of iron accumulation that occurs in neurodegenerative diseases. Therefore, the hippocampus was chosen to start with for further molecular biology measurement. The mice received deep pentobarbital sodium anesthesia and were then sacrificed by cervical dislocation. The inflammatory cytokines were measured 6 h after the microinjection of LPS, as that is the peak of the pro-inflammatory cytokines increase induced by LPS, as well as the onset of the further inflammatory cascade, as described in our previous study [13]. Six hours after LPS microinjection, the hippocampi of five mice in each group were obtained for enzyme-linked immunosorbent assay (ELISA), and the remaining brain samples were obtained 24 h after LPS treatment for all other molecular biology measurements, as that is consistent with the changes of behavioral performance. Five whole brains of mice in each group were fixed in 4% paraformaldehyde for at least 48 h, and then cryoprotected in 30% sucrose at 4 °C for an additional 48 h for Iba-1 immunofluorescence staining, TUNEL staining, and Nissl staining. The hippocampi of three mice from each group were postfixed overnight at 4 °C using 2.5% glutaraldehyde for mitochondrial transmission electron microscopy (TEM). The hippocampal tissue of the remaining mice was preserved at −80 °C for western blotting, commercial assay kits, and iron content measurement.

### 2.8. Immunofluorescence Staining

Paraffin-embedded coronary brain sections were examined using immunofluorescence staining 24 h after LPS microinjection (*n* = 4). After post-fixation and dehydration using a concentration gradient, the brains were cut into 15-μm-thick sections using a Leica RM2016 slicer. Paraffin-embedded sections were treated with xylene twice for 15 min each time. They were dehydrated using two 5-min rounds of treatment with pure ethanol, and then washed with distilled water. Antigen retrieval was performed in a microwave oven. Then, the sections were washed three times with PBS (pH 7.4). Subsequently, the sections were incubated in 3% BSA for 30 min to block non-specific epitopes. The slides were incubated with the primary antibody (rabbit anti-Iba-1: 1:500; Wako, Japan) overnight at 4 °C. Subsequently, the slides were incubated with goat anti-rabbit antibodies (1:2000; Cell Signaling Technology, Danvers, MA, USA) for 50 min at 22 °C in the dark. The slides were then incubated with mounting medium with DAPI (#104139; abcam, Cambridge, UK) for 10 min. The total number of Iba-1-positive microglia in the hippocampal DG was counted via fluorescence microscopy (Olympus America, Melville, NY, USA).

### 2.9. TUNEL Staining

TUNEL staining was performed to measure cell death in the DG region of the hippocampus 24 h after LPS microinjection (*n* = 4) according to the manufacturer’s instructions (In Situ Cell Death Detection Kit, Roche Applied Science, Mannheim, Germany). Briefly, brain sections were incubated with the permeabilization solution and TUNEL reaction mixture. Then, they were stained with DAPI. The sections were imaged using a fluorescence microscope and the number of TUNEL-positive cells in the hippocampal DG was counted using fluorescence microscopy at a ×200 magnification.

### 2.10. Nissl Staining

The morphological changes in DG hippocampal neurons were observed using Nissl staining 24 h after LPS microinjection (*n* = 5). The experimental procedure was performed in strict accordance with the manufacturer’s instructions (Nissl staining kit, G1036, Servicebio, Wuhan, China). Observers blinded to the treatment groups counted the total number of Nissl-positive neurons in the DG region under a light microscope (BX5; Olympus, Tokyo, Japan).

### 2.11. TEM

Hippocampi were postfixed using 2.5% glutaraldehyde 24 h after LPS microinjection (*n* = 3) and cut into 50-µm-thick coronal sections. TEM was performed as described previously [25]. Mitochondrial fragmentation and accumulation around the nucleus were analyzed as described by Anja et al. [26]. Briefly, the classifications were as follows: category I that contains an elongated mitochondrial network; category II that contains fragmented but evenly distributed mitochondria; and category III that contains mainly fragmented mitochondria.

### 2.12. Detection of Proinflammatory Cytokines

The levels of tumor necrosis factor-α (TNF-α) and interleukin-6 (IL-6) were examined using the TNF-α and IL-6 uncoated ELISA assay kits (ThermoFisher, Vienna, Austria) 6 h after LPS microinjection (*n* = 5). The hippocampi were homogenized in RIPA lysis buffer (Servicebio, Wuhan, China), and the tissue supernatant was extracted. The next steps were performed in accordance with the manufacturer’s instructions. The concentrations of TNF-α and IL-6 were expressed in pg/mL.

### 2.13. MDA, LPO, and GSH Concentrations and SOD Activity Assay

Malondialdehyde (MDA) levels and lipid peroxidation (LPO) are well-established indicators of lipid peroxidation. Glutathione (GSH) is an important antioxidant that can scavenge lipid peroxides through Gpx4 and inhibit ferroptosis. Superoxide dismutase (SOD) is the most important endogenous scavenger of reactive oxygen species (ROS) and maintains metabolic balance in organisms. We used commercial assay kits (Nanjing Jiancheng, Nanjing, China) to measure the MDA, LPO, GSH, and SOD levels 24 h after LPS microinjection (*n* = 6). The experimental procedures were performed in accordance with the manufacturers’ instructions.

### 2.14. Detection of Iron Content

The total iron content in the hippocampus was detected in accordance with the manufacturer’s instructions (Iron Assay Kit, Nanjing Jiancheng, Nanjing, China) 24 h after LPS microinjection (*n* = 6). Briefly, the hippocampi were weighed and homogenized, and the tissue supernatant was extracted. The supernatant was added to a mixed iron chromogenic solution according to the manufacturer’s instructions. The OD value of each tube was then examined at a wavelength of 450 nm by a microplate reader (SpectraMax i3x; Molecular Devices, San Jose, CA, USA).

### 2.15. Western Blotting

Hippocampal tissue was used for western blotting analysis 24 h after LPS microinjection (*n* = 6). Briefly, 50 μg of protein was separated on a 4–12% SDS-PAGE gel and transferred to a PVDF membrane. The primary antibodies were anti-Gpx4 (1:100; Senta Cruz, TX, USA), anti-MtFt (1: 250; Gene Tex, CA, USA), anti-Fth (1:1000; Cell Signaling Technology, Danvers, MA, USA), anti-xCT (1:1000; Cell Signaling Technology, Danvers, MA, USA), anti-TF (1:1000; Abcam, Cambridge, UK), anti-GAPDH (1:1000; Cell Signaling Technology, Danvers, MA, USA), and anti-β-actin (1:1000; Cell Signaling Technology, Danvers, MA, USA). The membranes were incubated with anti-rabbit or anti-mouse fluorophore-conjugated secondary antibodies (LI-COR, Lincoln, NE, USA) for 1.5 h, and bands were detected using an Odyssey scanner (LI-COR, Lincoln, NE, USA). The images were analyzed using HIN ImageJ software.

### 2.16. Statistical Analysis

All data were analyzed by observers blinded to the experimental protocols and group using GraphPad Prism (Version 8.0, San Diego, CA, USA). The results were shown as the mean ± standard error (SE). Two-way ANOVA with Tukey’s post hoc test was used for comparing differences among the four experimental groups. For the MWM test, the data were analyzed using a two-way repeated-measures ANOVA with Bonferroni’s post hoc test. Values with *p* < 0.05 were considered statistically significant.

## 3. Results

### 3.1. Liproxstatin-1 Attenuated LPS-Induced Cognitive Impairment

**Figure 1:**
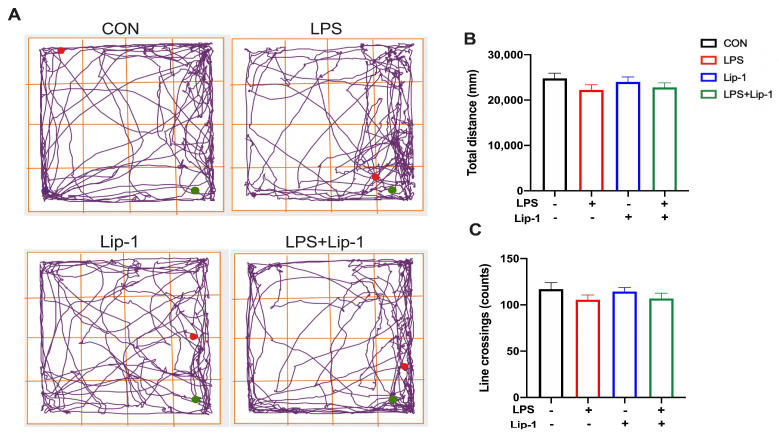
LPS and liproxstatin-1 did not affect the spontaneous activity of mice in the open field test. (**A**) Representative moving trajectories of mice in the open arena during the 8 min. The green spot presents the start of mice movement, and the red spot presents the end. (**B**) Total distance, (**C**) line crossings are expressed as mean ± SE (*n* = 12).

We have previously demonstrated that the intracerebroventricular administration of LPS induced cognitive deficits in the male C57BL/6 mouse models [13,27]. Therefore, we examined the protective effects of liproxstatin-1 on memory deficits induced by LPS microinjection in this model. In the OF test, there were no significant differences in the line crossings and the total distance (*p* = 0.72 and 0.54 for line crossings and total distance; Figure 1B,C) between the four groups 24 h after LPS treatment, suggesting that the impaired performance differences in behavioral test among the groups were not due to the differences in spontaneous locomotor activity.

**Figure 2:**
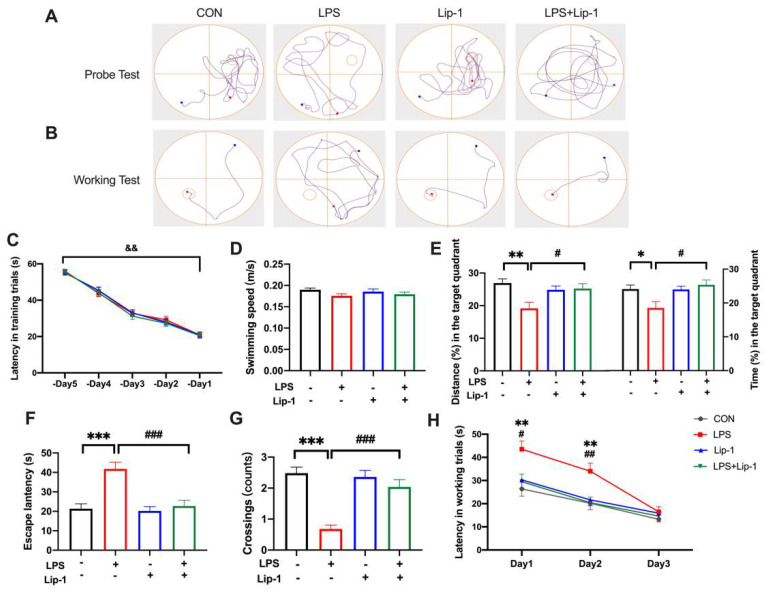
Liproxstatin-1 attenuated behavioral performance in MWM test after exposure to LPS in mice. After five consecutive days of acquisition phase in MWM test, the mice received LPS (or aCSF) microinjection. (**A**) Representative trajectories of mice in four groups in the probe test. The small circle presents the site of platform, which was removed during the probe test. (**B**) Representative trajectories of mice to the new platform in spatial working memory test, in which the hidden platform was randomly changed to another quadrant (the third quadrant in this representative trajectory). (**C**) Latency to the platform over the five training days. (**D**) Swimming speed, (**E**) the percentage of distance and time at the platform-site quadrant, (**F**) latency to the platform, (**G**) platform-site crossings during the probe test. (**H**) Latency to the new platform during spatial working memory test on Day 1, 2, and 3 after LPS treatment. Data are expressed as mean ± SE (*n* = 25 for probe test and *n* = 17 for working memory test). * *p* < 0.05, ** *p* < 0.01, *** *p* < 0.001 vs. CON group; ^#^ *p* < 0.05, ^##^ *p* < 0.01, ^###^ *p* < 0.001 vs. LPS group; ^&&^ *p* < 0.01 -Day 1 vs. -Day 5.

During the acquisition phase of the MWM, the performance of the mice in locating the hidden platform improved with training. There were no significant differences in the latency to the platform among the groups for each day (Figure 2C). The probe test and working memory test were performed 24 h after LPS administration. During the probe test, no significant differences in swimming speed were observed among the groups, indicating that performance differences were not caused by differences in spontaneous activity or motor ability (*p* = 0.416; Figure 2D). The latency to reach the platform site was significantly higher in the LPS group than in the CON group, while it was lower in the LPS + Lip-1 group (*p* < 0.001; Figure 2F). This indicated that mice in the LPS group spent more time to reach the platform site, and suggested that their memory of strategy for using the references and route to the platform they learned from the training was inferior to the mice of the CON group, which was alleviated by liproxstatin-1. Likewise, the percentage of distance and time within the platform-site quadrant (*p* < 0.01, and *p* < 0.05 for the percentage of distance and time respectively; Figure 2E) and the number of platform-site crossings (0.68 ± 0.12 in LPS group vs. 2.04 ± 0.23 in LPS + Lip-1 group; *p* < 0.001; Figure 2G) were significantly lower in the LPS group than those in the CON group, further indicating that the intracerebroventricular LPS treatment blurred the memory of shortest path and platform site in mice. Additionally, these indicators were increased in the LPS + Lip-1 group compared to the LPS group. Similar results for latency to the platform were observed on days 1 and 2 of the working memory test. The latency to the platform site was significantly higher in the LPS group than in the CON group, while it was decreased in the LPS + Lip-1 group compared to the LPS group. The working memory test reflected the short-term learning and memorizing ability for searching the new platform. These decreased after the LPS treatment in mice, which was attenuated in the LPS + Lip-1 group. There were no significant differences in the above parameters among the groups on day 3 (*p* < 0.01 for days 1 and 2; Figure 2H).

**Figure 3:**
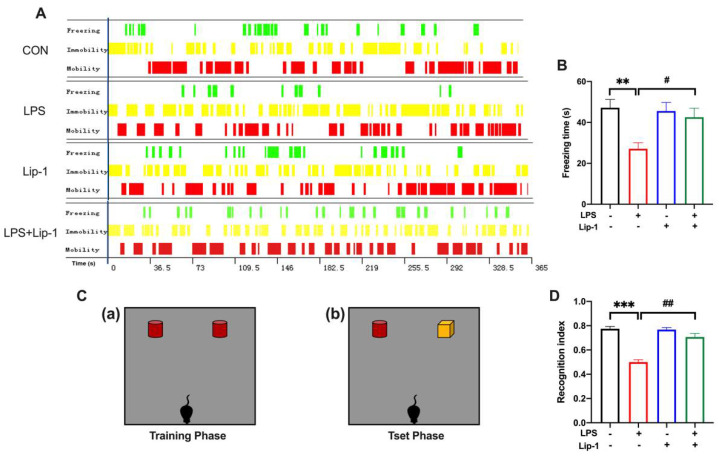
Liproxstatin-1 improved the behavioral performance in the contextual fear conditioning and novel objective recognition test after exposure to LPS in mice. (**A**) Representative time distribution of freezing, immobility and mobility in the four groups of mice. (**B**) The freezing time during the contextual fear conditioning test (*n* = 12). (**C**) The schematic diagram for the (**a**) training phase and (**b**) test phase in the novel objective recognition test. (**D**) The recognition index in the test phase (*n* = 12). Data are expressed as mean ± SE. ** *p* < 0.01, *** *p* < 0.001 vs. CON group; ^#^ *p* < 0.05, ^##^ *p* < 0.01 vs. LPS group.

The contextual fear conditioning test assessed hippocampus-dependent associative learning and memory. Mice expressed a fear response (freezing) after connecting the neutral stimulus with an aversive stimulus (foot shock). Freezing was a defensive behavior of mice, and it is considered a reliable indicator for evaluating fear behavior. The freezing time in the contextual test was significantly lower in the LPS group than in the CON group, while it was higher in the LPS + Lip-1 group (*p* < 0.05; Figure 3B). This indicated that mice in the CON group expressed obvious context-related fear behavior, whereas the mice in the LPS group had a weakened memory of the connection between the context and the fear. These results suggested that LPS impaired the memory of recalling the association between the context and the fear, which was alleviated by liproxstatin-1.

The NOR test was used to assess the recognition memory in the mice in our study. Due to the innate preference for novelty, if the mice recognized the familiar objects they had seen in the environment, they would spend more time exploring the new object. A significantly decreased recognition index was observed in mice in the LPS group compared with the CON group, while it was increased in the LPS + Lip-1 group (*p* < 0.001; Figure 3D). These results showed that mice in the LPS group tended to spend similar amounts of time exploring the new and old objects, and the recall was weakened. However, liproxstatin-1 relieved the LPS-induced impairment of recognition memory.

To sum up, the above results suggested that LPS microinjection induced cognitive impairment, including spatial-dependent memory, contextual-dependent memory, and object recognition memory, which is ameliorated by the ferroptosis inhibitor liproxstatin-1.

### 3.2. Liproxstatin-1 Suppressed Microglial Activation and Alleviated LPS-Induced Inflammation

Iron has been reported to be involved in LPS-induced neuroinflammation in mice [13]. Hence, we explored whether ferroptosis could participate in the development of neuroinflammation. Thus, we tested microglial activation and the concentration of two classic proinflammatory cytokines (IL-6 and TNF-α) in the hippocampus after LPS treatment.

**Figure 4:**
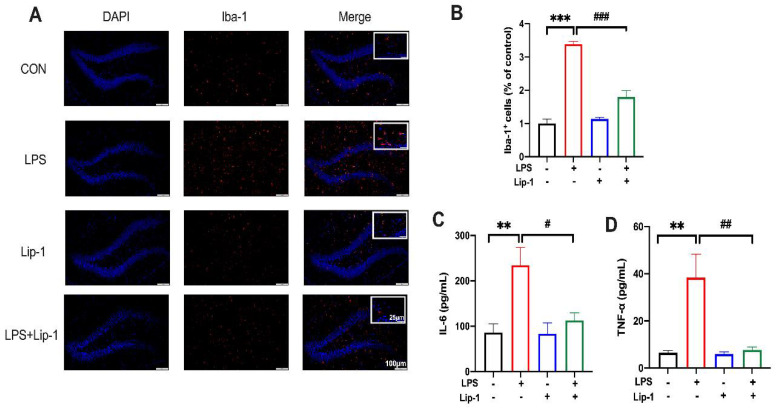
Liproxstatin-1 alleviated LPS-induced microglial activation and inflammatory cytokine accumulation. (**A**) Representative images of Iba-1 labeled activated microglia in the hippocampal DG region (scale bar = 100 μm) and magnified views (scale bar = 25 μm). (**B**) The numbers of Iba-1^+^ cells in the hippocampal DG region (*n* = 4). (**C**,**D**) Levels of IL-6 and TNF-α in the hippocampus (*n* = 5). Data are expressed as mean ± SE. ** *p* < 0.01, *** *p* < 0.001 vs. CON group; ^#^ *p* < 0.05, ^##^ *p* < 0.01, ^###^ *p* < 0.001 vs. LPS group.

Immunofluorescence analysis showed that microglial activation, indicated by Iba-1 staining in the hippocampal DG, was significantly stronger in the LPS group than in the CON group. However, data from the LPS + Lip-1 group showed that liproxstatin-1 significantly decreased the number of Iba-1-positive cells (*p* < 0.001; Figure 4B).

ELISA results showed that LPS caused a significant increase in IL-6 (*p* < 0.01; Figure 4C) and TNF-α levels (*p* < 0.01; Figure 4D) in the hippocampus (LPS group vs the CON group). However, liproxstatin-1 significantly attenuated this LPS-induced increase in IL-6 (*p* < 0.05; Figure 4C) and TNF-α levels (*p* < 0.01; Figure 4D).

Collectively, these data indicated that liproxstatin-1 alleviated LPS-induced microglial activation and inflammatory cytokine release in the hippocampus.

### 3.3. Liproxstatin-1 Attenuated the LPS-Induced Increase in MDA and LPO Levels and Decreased SOD Activity and GSH Concentrations and Improved Mitochondrial Injury in Hippocampal Neurons

**Figure 5:**
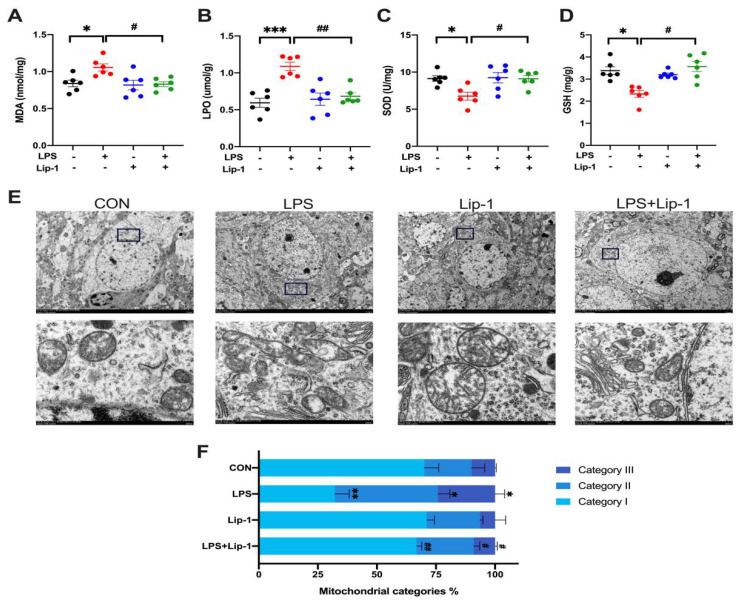
Liproxstatin-1 ameliorated oxidative stress, lipid peroxidation and hippocampus neuronal mitochondria injury caused by LPS. (**A**) MDA levels, (**B**) LPO levels, (**C**) SOD activities and (**D**) GSH contents in hippocampus (*n* = 6). (**E**) Transmission electron microscopy images of hippocampus neuronal mitochondria from each group (scale bar = 5 µm for above and 500 nm for below). (**F**) The percentage of different types of hippocampus neuronal mitochondria: category I (containing a network of elongated mitochondria), category II (containing fragmented but evenly distributed mitochondria), category III (containing mainly fragmented mitochondria) (*n* = 3). Data are expressed as mean ± SE. * *p* < 0.05, ** *p* < 0.01, *** *p* < 0.001 vs. CON group; ^#^ *p* < 0.05, ^##^ *p* < 0.01 vs. LPS group.

Lipid peroxide deposition and lipid oxidative stress are critical steps in the development of ferroptosis [28,29]. Our results showed that MDA and LPO levels in the LPS group were significantly higher than those in the CON group (*p* < 0.05 and *p* < 0.001 for MDA and LPO respectively; Figure 5A,B), and SOD and GSH contents were significantly lower (*p* < 0.05 for SOD and GSH; Figure 5C,D). These changes were alleviated by liproxstatin-1. There was no significant difference between the CON group and the Lip-1 group.

Ferroptosis causes changes in mitochondrial morphology, including shrinkage of the mitochondrial membrane and reduction or disappearance of mitochondrial cristae [30]. As shown in the results, the hippocampal mitochondria had a normal morphology in the CON group. In the LPS group, many mitochondrial outer membranes were broken, and the cristae had decreased or disappeared. In the LPS + Lip-1 group, the mitochondria were uniformly distributed, and the outer membranes were more complete. As shown in Figure 5F, the number of type I mitochondria was significantly lower, while the number of type II and type III mitochondria was significantly higher in the LPS group than in the CON group (*p* < 0.01, *p* < 0.05, and *p* < 0.05 for type I, type II, and type III respectively; Figure 5F). Liproxstatin-1 increased the number of type I mitochondria and decreased the number of type II and III mitochondria (*p* < 0.01, *p* < 0.05, and *p* < 0.05 for type I, type II, and type III; Figure 5F).

Collectively, our data showed that LPS microinjection induced oxidative stress and lipid peroxidation, and increased the number of damaged mitochondria, which can be alleviated by liproxstatin-1.

### 3.4. Liproxstatin-1 Attenuated LPS-Induced Iron Deposition and Altered the Expression of Ferroptosis-Related Proteins in the Hippocampus

**Figure 6:**
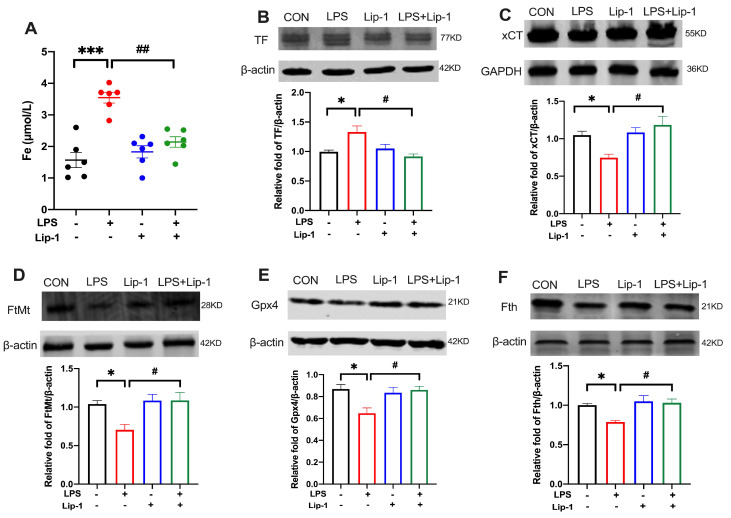
Liproxstatin-1 attenuated the iron deposition and altered expression of proteins related to ferroptosis in the hippocampus of mouse model of LPS microinjection. (**A**) The level of iron content in hippocampus (*n* = 6). (**B**–**F**) Cropped gels and blots showing the protein expression and the graph of the protein expression of the (**B**) TF, (**C**) xCT, (**D**) FtMt, (**E**) Gpx4 and (**F**) Fth (*n* = 6). Data are expressed as mean ± SE. * *p* < 0.05, *** *p* < 0.001 vs. CON group; ^#^ *p* < 0.05, ^##^ *p* < 0.01 vs. LPS group.

Iron accumulation can cause tissue damage, and disturbances in iron metabolism are essential for lipid peroxide accumulation and initiation of the ferroptosis pathway [31]. Our data showed a significant increase in iron content in the LPS group (*p* < 0.001; Figure 6A), which was ameliorated by liproxstatin-1 (*p* < 0.01; Figure 6A).

The expression levels of ferroptosis-related proteins (TF, xCT, FtMt, Gpx4, Fth) were determined by western blot. The levels of xCT, FtMt Gpx4, and Fth were lower in the LPS group than in the CON group, but in the LPS + Lip-1 group, liproxstatin-1 attenuated this change (*p* < 0.05 for xCT, FtMt, Gpx4, Fth; Figure 6C–F). The level of TF was higher in the LPS group than in the CON group (*p* < 0.05; Figure 6B) but it was lower in the LPS + Lip-1 group than in the LPS group (*p* < 0.05; Figure 6B).

### 3.5. Liproxstatin-1 Alleviated LPS-Induced Neuronal Damage in the Hippocampus

**Figure 7:**
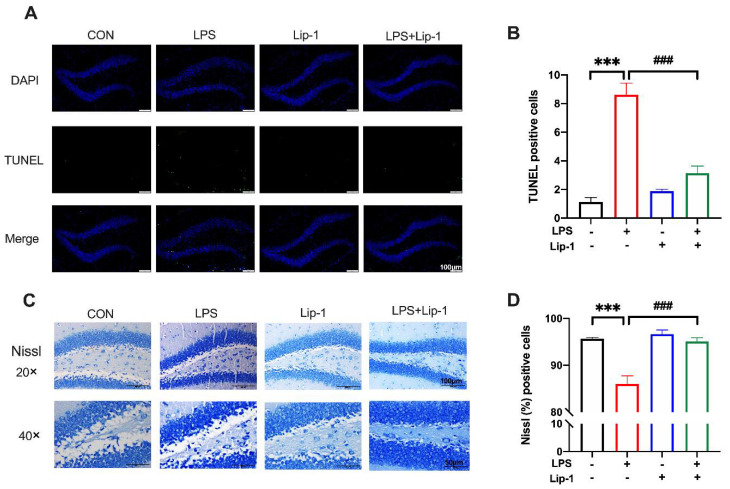
Liproxstatin-1 improved LPS-induced hippocampus neuronal injury. (**A**) TUNEL staining demonstrated cell death in the DG region of the hippocampus in four groups (scale bar = 100 μm). (**B**) The number of TUNEL-positive cells (*n* = 4). (**C**) Nissl staining showed morphological neuronal changes in the DG region of hippocampus (scale bar = 100 μm for above and 50 μm for below). (**D**) The percentage of Nissl-positive cells (*n* = 5). Data are expressed as mean ± SE. *** *p* < 0.001 vs. CON group; **^###^** *p* < 0.001 vs. LPS group.

TUNEL staining demonstrated neuronal death in the hippocampal DG region. Compared with the CON group, the TUNEL-positive cells number was significantly higher in the LPS group (*p* < 0.001; Figure 7B). However, the number of TUNEL-positive cells was significantly lower in the LPS + Lip-1 group (*p* < 0.001; Figure 7B). Nissl staining results revealed the morphological changes in neurons in the hippocampus DG region. As shown in Figure 7C, the Nissl-positive neurons were morphologically intact, while injured neurons had shrunken cell bodies. The proportion of Nissl-positive cells was decreased in the LPS group compared with that in the CON group (*p* < 0.001; Figure 7D), while it was higher in the LPS + Lip-1 group than in the LPS group (*p* < 0.001; Figure 7D).

## 4. Discussion

Our study indicated that LPS microinjection can cause inflammation, oxidative stress, and severe ferroptosis in the mouse hippocampus, leading to impaired learning and memory performance. Treatment with the ferroptosis inhibitor liproxstatin-1 significantly alleviated this LPS-induced learning and memory impairment. The protective effect of liproxstatin-1 against memory impairment could be attributed to the neuroprotective effects of alleviating oxidative stress, reducing inflammation response and iron accumulation, and attenuating mitochondrial damage, thus reducing the occurrence of ferroptosis and neural damage in the mouse hippocampus.

Although the inflammation-related cognitive dysfunction model was established years ago, so far, its detailed mechanisms are not yet fully understood [13,27]. Our previous study showed that during LPS-induced cognitive impairment, obvious iron deposition and metabolic imbalance occurred, as well as an increase in ROS levels, which is consistent with this study [13]. Abnormal iron homeostasis occurs in a variety of cognitive disorders [32,33], but the means by which iron participates in regulating LPS-induced cognitive impairment has rarely been studied. Ferroptosis is a newly discovered form of cell death that is characterized by non-apoptotic, iron-dependent, and severe oxidative damage, and has been linked to many neurodegenerative diseases [14,34,35,36,37,38,39,40,41]. Hippocampal iron deposition and the accompanying oxidative stress response during LPS-induced cognitive impairment are consistent with the timing and mechanisms of ferroptosis. Therefore, this study explored the effect of ferroptosis on neuroinflammation and inflammation-related PND using the ferroptosis inhibitor liproxstatin-1 in a mouse model of LPS-induced memory impairment.

The MWM test, contextual fear conditioning test, and NOR test are commonly used to evaluate learning and memory in rodents [24,42,43]. The results of LPS-induced memory deficits were in agreement with previous studies [13,22,27,44], confirming the success of the LPS-induced cognitive impairment model. We further observed liproxstatin-1-alleviated LPS-induced learning and memory impairments. Consistent with our findings, studies reported that liproxstatin-1 ameliorated cognitive dysfunction in rodent models, including traumatic brain injury and subarachnoid hemorrhage, by improving ferroptosis associated with neurological results, such as lipid peroxidation, iron deposition, and ferroptosis-related protein [45,46]. These findings suggest that ferroptosis plays a vital role in the process of LPS-induced cognitive impairment, and liproxstatin-1 could have a potential therapeutic effect against such cognitive dysfunction.

Neuroinflammation is an important pathological characteristic of neurodegenerative disease, and is observed in multiple animal models of cognitive impairment [47,48,49]. Our study showed that an LPS microinjection can activate microglia and increase the levels of the proinflammatory cytokines IL-6 and TNF-α. Treatment with liproxstatin-1 attenuated this inflammatory response, indicating that ferroptosis is involved in neuroinflammation. In the LPS-induced neuroinflammatory environment, immunogenic molecules can “activate” microglia, causing the release of pro-inflammatory cytokines and subsequent neuronal damage [50]. An oxidative burst represents one of the earliest biochemical events following the inflammatory activation of microglia. Microglial activation leads to the constant release of proinflammatory cytokines and to the generation of ROS, which can contribute to neurodegenerative diseases [51,52]. Meanwhile, inflammatory mediators, such as IL-6, can directly trigger the production of hepcidin, a key regulator of iron metabolism. Hepcidin catalyzes the internalization and degradation of ferroportin, the sole iron exporter, resulting in iron accumulation, which is involved in the pathophysiology of multiple neurodegenerative diseases. Ferroptosis is characterized by iron-dependent oxidative stress and leads to the release of damage associated molecular patterns (DAMPs) and immunogenic lipid metabolites [53,54], which can enhance inflammation [35]. In ferroptosis, ROS has been reported to promote the inflammatory response and regulate the levels of different inflammatory cytokines [55,56,57], which is consistent with our findings.

Ferroptosis is a regulated form of necrotic cell death, and is dependent on oxidative stress and lipid peroxidation. Our data suggested that liproxstatin-1 alleviated LPS-induced oxidative stress and lipid peroxidation by increasing the content of SOD and decreasing MDA and LPO levels. The brain has a high metabolic rate and relatively low antioxidant defenses [58]; hence, it is vulnerable to oxidative stress, which is mainly caused by ROS overproduction [59]. Oxidative damage in the brain mainly manifests as lipid peroxidation. Excess ROS can contribute to the accumulation of lipid peroxidation and induce oxidative stress. SOD is the most important endogenous scavenger of ROS. Further, MDA is the product of lipid peroxidation by the oxygen free radicals and polyunsaturated fatty acids on biofilm. Therefore, SOD and MDA are indicators of oxidative stress and lipid peroxidation, respectively. Antioxidant enzymes, such as GPXs and SODs, represent the main antioxidant defense system of the CNS. These enzymes catalyze the transformation of ROS and ROS byproducts into stable nontoxic molecules, thereby counteracting oxidative stress-induced cellular damage and maintaining redox homeostasis. High levels of lipid peroxidation drive ferroptosis [60]. Bao et al. [21] reported that liproxstatin-1 attenuates ferroptosis by decreasing ROS levels and lipid peroxidation in the hippocampus, ameliorating cognitive dysfunction in AD mice. Liu et al. [61] also reported that ferroptosis participates in LPS-induced acute lung injury by aggravating oxidative stress and lipid peroxidation in the inflammatory environment. Cui et al. [62] demonstrated both in vitro and in vivo that ferroptosis could be up-/down-regulated by enhancing/suppressing lipid peroxidation with over-expression/knockdown of acyl-CoA synthetase long-chain family member 4 (ACSL4), and these changes were consistent with the neuroinflammation. Overall, these results are in accordance with our findings, showing that oxidative stress and lipid peroxidation may be involved in LPS-induced ferroptosis, and that liproxstatin-1 could alleviate ferroptosis in LPS-treated mice through the attenuation of this process.

On a morphological basis, ferroptosis mainly causes changes in mitochondrial morphology [41,63]. In our study, LPS microinjection destroyed the outer mitochondrial membranes, caused the decreased or disappearance of mitochondrial cristae, and increased the number of damaged mitochondria. However, the mitochondria were uniformly distributed in the LPS + Lip-1 group; their outer membranes were more complete, and the number of damaged mitochondria was lower. Mitochondria play a crucial role in energy metabolism, cell death regulation, and cell signaling. In an inflammatory microenvironment, mitochondria exhibit ROS overproduction, which promotes inflammation by causing genomic instability and exacerbating inflammatory pathways. Oxidative damage impairs oxidative phosphorylation, leading to mutations in mitochondrial and nuclear DNA, which further promotes mitochondrial ROS production. This leads to a “vicious circle” between the mitochondria, reactive oxygen species, and inflammation [30,64,65]. In particular, the loss of mitochondrial integrity and function is considered a hallmark of oxidative neuronal death [26]. Mitochondrial shrinkage and destruction in the hippocampus have been reported as signs of ferroptosis in AD mouse models, and have been reported to be attenuated by liproxstatin-1 [21]. Wang et al. [66] showed mitochondrial morphological changes, including smaller volume, higher electron density, and disrupted mitochondrial cristae, in the erastin-induced ferroptosis in HT22 cells, and that the morphological changes were accompanied by an increase of lipid peroxidation and the activation of NF-κB signaling. These results suggest that mitochondria could be the crucial part and key position in the process by which neuroinflammation induces oxidative stress and ferroptosis, further aggravating the inflammatory response and destructive changes, and forming a vicious circle. Mitochondrial dysfunction and morphological abnormalities may further aggravate oxidative stress and lipid peroxidation, further contributing to ferroptosis and cognitive dysfunction. In turn, liproxstatin-1 can attenuate mitochondrial damage by ameliorating ferroptosis and oxidative stress.

The brain has the second-highest concentration of iron in the body after the liver. Functionally, iron is involved in many fundamental biological cellular processes in the brain, including mitochondrial respiration, antioxidant enzyme activity, and neurotransmitter synthesis and metabolism [67]. Neurodegenerative diseases are associated with complex mechanisms of cell death and multiple pathways that involve excessive iron deposition in different brain regions [17]. Iron metabolism disorders can cause neurotoxicity and neurological impairment through iron-induced free radical damage, inflammatory responses, and mitochondrial dysfunction [68]. Collectively, iron could play a key role in the onset and progression of neurodegenerative diseases [32,69]. In our experiments, LPS increased the iron content in the hippocampus. Iron is required for the accumulation of lipid peroxides and the execution of ferroptosis [29,41]. Transferrin is an iron carrier protein that can be transported into cells via receptor-mediated endocytosis [70,71]. Transferrin loaded with iron binds to the transferrin receptor and is transported across the cell membrane. On the other hand, ferritin, as the storage of iron in cells, consists of 24 heavy (Fth) and light (Ftl) subunits, and can store up to 4500 iron atoms. In addition, Fth shows ferroxidase activity, catalyzing the conversion of Fe^2+^ to Fe^3+^ and storing it inside the ferritin nanocage [45]. Our results showed that transferrin levels were increased and Fth levels were decreased in the LPS-induced neuroinflammation. This suggested that iron metabolism disorders and iron accumulation were involved in LPS-induced neuroinflammation. This was in line with our previous findings, which showed that the iron chelator deferoxamine could reduce iron deposition and metabolic iron dysfunction and ameliorate inflammation [13]. Iron deposition leads to ROS production and contributes to oxidative stress via the Fenton reaction, further promoting lipid peroxidation, which is the key contributor to ferroptosis. These processes eventually result in cell death, which was reported in vivo and in vitro [33,72,73,74]. As shown in our study, liproxstatin-1, a ferroptosis inhibitor, can prevent lipid peroxidation and alleviate the abovementioned changes in iron metabolism. These effects of liproxstatin-1 on iron metabolism may contribute to its neuroprotective effects against oxidative stress and inflammation.

Mitochondrial ferritin (FtMt) is a key mitochondrial iron storage protein. Its ferroxidase activity facilitates the catalyzing of the conversion from Fe^2+^ to Fe^3+^, and FtMt further stores the Fe^3+^ inside its spherical shell [75,76,77]. FtMt can protect cells from iron-dependent oxidative damage [78,79,80]. Our results showed that FtMt expression was reduced in the LPS group, while liproxstatin-1 prevented this reduction. Mitochondrial iron is an important mediator of the oxidative stress response. As a key mitochondrial iron storage protein, the protective role of FtMt in neurodegenerative diseases has also been reported in AD and Parkinson’s disease models [81,82,83]. Wang et al. [84] showed that the overexpression of FtMt in neuroblastoma SH-SY5Y cells could significantly inhibit ferroptosis by storing excess iron elevation and decreasing ROS production. Additionally, the protective effect of FtMt was also observed in drosophila. FtMt may serve this function by suppressing the expression of voltage-dependent anion channels (VDACs) and the activation of nicotinamide adenine dinucleotide phosphate (NADPH)-dependent oxidase 2 (NOX2). Therefore, the decrease of FtMt expression in the LPS-induced neuroinflammatory environment may participate in the mechanism by which inflammation leads to the iron accumulation, oxidative stress, and the occurrence of ferroptosis in mitochondria.

The transmembrane cystine-glutamate antiporter system Xc^−^, which includes a light chain xCT (SLC7A11) and a heavy chain 4F2 (SLC3A2), is involved in the ferroptosis-signaling cascade [85]. The inhibition of system Xc^−^ can deplete the intracellular cysteine pool [41]. Cysteine plays a critical role in cells and acts as a building block for GSH biosynthesis in ferroptosis. GSH acts as a Gpx4 cofactor to maintain Gpx4 levels by exchanging glutamate and cystine through the cysteine–glutamate antiporter system Xc^−^. Gpx4 is a GSH-dependent enzyme that reduces lipid hydroperoxides (L-OOH) to lipid alcohols (L-OH) [86], thereby preventing the Fe^2+^-dependent formation of toxic lipid ROS. Our results revealed that LPS treatment decreases xCT, GSH, and Gpx4 levels, while liproxstatin-1 alleviates these changes. Both xCT and Gpx4 are considered to be central regulators of ferroptosis, and decreases of xCT and Gpx4 are considered to be markers of ferroptosis [87,88,89] that can be prevented by ferroptosis inhibitors [45,61], these results were also demonstrated in vitro [90,91,92]. These findings are consistent with the results of our study. Overall, the downregulation of xCT, GSH, and Gpx4 in an inflammatory environment induces ferroptosis by activating lipid ROS and peroxidation. While, liproxstatin-1 ameliorates lipid oxidative stress and lipid peroxidation, restores the expression of xCT, GSH, and Gpx4, and inhibits ferroptosis. The detailed mechanism of ferroptosis on neurodegenerative diseases remains to be explored in vitro in the future.

Cell death is necessary for maintaining the growth, development, and activity of an organism. Ferroptosis, an iron-dependent form of regulated cell death caused by iron-mediated lipid peroxidation and oxidative stress, is observed in many neurodegenerative diseases. Our results indicated that LPS could induce neuronal damage and death, while liproxstatin-1 could attenuate its effects. These results are consistent with pervious evidence showing that liproxstatin-1 and ferrostatin-1, another ferroptosis inhibitor, can ameliorate the neuronal death induced by Aβ1–42 in AD models [21].

**Figure 8:**
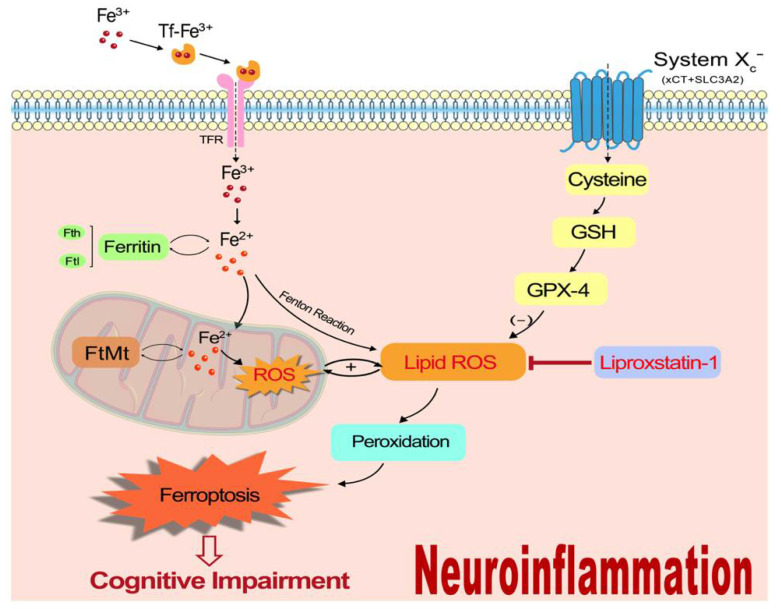
A schematic illustration of the proposed mechanism for ferroptosis to participate in neuroinflammation-induced memory impairment.

This study mainly indicated that ferroptosis could participate in the mechanism underlying LPS-induced cognitive dysfunction by taking part in iron metabolism imbalance, motivating oxidative stress, activating the inflammatory cascade response, and contributing further to neuronal damage (Figure 8). Our previous studies showed the important role of iron metabolism and the protective effect of iron chelator deferoxamine in neuroinflammation-induced cognitive dysfunction [13,93], and this study further demonstrated that ferroptosis may be the underlying mechanism for the effect of iron regulating LPS-induced memory impairment. It provides a new strategy for the inflammation-related cognitive disorder without causing the potential adverse effect of iron chelator in clinical application. However, this study is not devoid of limitations. The specific molecular and genetic mechanisms by which ferroptosis contributes to neuroinflammation-induced PND could not be uncovered, as these were beyond the scope of this study. Moreover, other forms of cell death, such as necroptosis, autophagy, and pyroptosis, could also be pro-inflammatory. Whether ferroptosis acts in concert with these processes remains to be examined.

## 5. Conclusions

In summary, our study indicates that ferroptosis plays a very important role in LPS-induced cognitive dysfunction, and suggests that ferroptosis may be the key to the means by which iron regulates inflammation-related PND. The ferroptosis inhibitor liproxstatin-1 ameliorates LPS-induced cognitive dysfunction by attenuating oxidative stress, subsiding inflammatory response, protecting mitochondrial function, and preventing neuronal damage. This study may provide novel potential ferroptosis-based therapeutic targets for PND.

## Data Availability

Data presented in this study are available on request from the corresponding author.
